# Insufficient Evidence for Rare Activation of Latent HIV in the Absence of Reservoir-Reducing Interventions

**DOI:** 10.1371/journal.ppat.1005679

**Published:** 2016-08-25

**Authors:** Alison L. Hill, Daniel I. S. Rosenbloom, Janet D. Siliciano, Robert F. Siliciano

**Affiliations:** 1 Program for Evolutionary Dynamics, Department of Mathematics, Department of Organismic and Evolutionary Biology, Harvard University, Cambridge, Massachusetts, United States of America; 2 Department of Biomedical Informatics, Columbia University Medical Center, New York, New York, United States of America; 3 Department of Medicine, Johns Hopkins University School of Medicine, Baltimore, Maryland, United States of America; 4 Howard Hughes Medical Institute, Chevy Chase, Maryland, United States of America; University of North Carolina at Chapel Hill, UNITED STATES

A priority for HIV cure research is measuring latent infection that can fuel viral recrudescence if patients cease antiretroviral therapy (ART). One important quantity that is difficult to measure is the rate at which latently infected cells activate, giving rise to spreading infection [1]. Pinkevych et al. recently estimated this rate by analyzing several clinical cohorts and concluded that one cell activates every 6 days [2]. This rate is 24-fold lower than our previous estimate of 4 cells/day [1], resulting in far more optimistic predictions for the prospects of reservoir-reducing therapy. We question their estimation approach and suggest that a higher rate is likely for most infected individuals.

The main problem with the analysis is that it treats all participants as identical, with a homogeneous virus population that activates and grows at the same rate. The only variation between participants considered is the (random) timing of viral activation. After treatment interruption, it is common to see one person rebound after two weeks, and another after three weeks. By their model, the first person's virus must have activated one week before the second person's virus did. If the activation rate were high, this would be extremely unlikely. The authors conclude that viral activation occurs about once per week, not multiple times per day as previously proposed [1,3–5]. Below, we offer an alternative explanation. We show that inter-person variation in viral activation or growth rates can explain the observed variation in rebound times. While Pinkevych et al. discuss the issue of interperson variation, they hypothesize that their fitted values properly estimate cohort averages. Our analysis below challenges this hypothesis: interperson variation does not merely lead to variation around a central estimate in activation rate but rather skews the estimate downward. We conclude by arguing that high activation rates are suggested by the weak correlation between preinterruption latent reservoir sizes and postinterruption viral rebound times.

In the Pinkevych et al. model, latently infected cells reactivate at a constant rate (*k*). Reactivated virus cannot spread until drug “washes out” following ART interruption. Viral activation occurs at a random, exponentially distributed time (average value 1/*k*) after washout. Plasma virus levels then grow exponentially from *V*_*0*_ at rate *r*. The proportion of individuals with viral rebound to detectable levels by a particular time can be calculated and compared to participant data to estimate parameters.

Pinkevych et al. use data from four cohorts [6–9] in which rebound times were recorded following treatment interruption: median rebound times were 8 to 14 days. They estimate that one activation occurs every 7.6, 6.3, 5.1, or 6.3 days for cohorts 1 through 4, respectively (equivalent to *k* = 0.13, 0.16, 0.20, 0.16/day). Following their model, we recreate fits to cohort 3 data in Fig 1A. We chose cohort 3 because it was the only study in which an actual estimate of rebound time was given, instead of just a time of first detectable value, which—depending on the frequency of sampling—could be off from the rebound time by up to a week. Cohort 3 therefore has a data point for each patient, not just for each sampling point. Results for additional cohorts are provided in S1 Text. In the lower panel, we provide viral load trajectories of 10 simulated participants, following their model but allowing for exponentially distributed activation events subsequent to the first (shown as randomly timed “jumps” in viral load).

**Fig 1:**
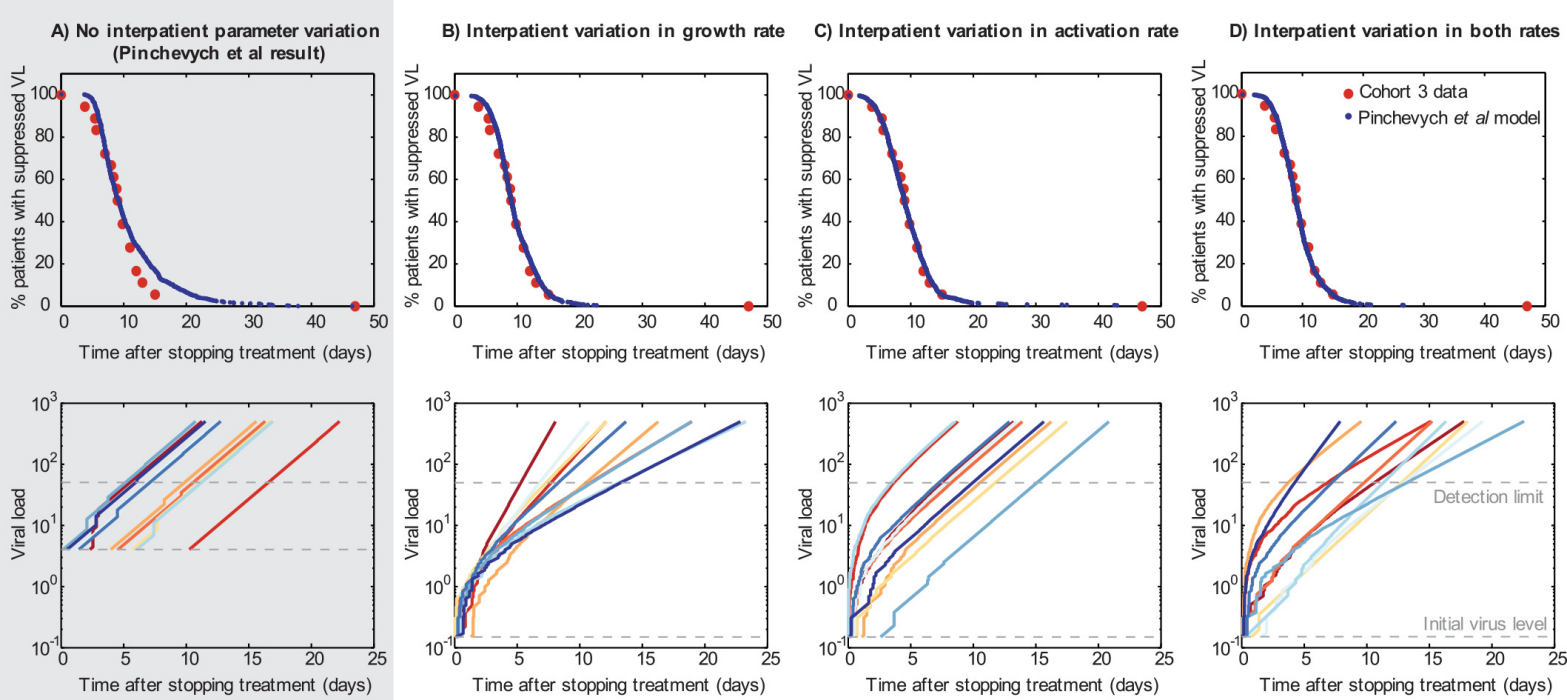
Alternatives to the Pinkevych et al. interpretation of rebound dynamics. Time-to-rebound data can be explained equally well by frequent reactivation in a realistically heterogeneous cohort (Fig 1B–D) as by rare reactivation in a homogeneous population (Fig 1A). Top row: Observed rebound times in “Cohort 3” [8] and best fits from models described in text. Bottom row: Representative rebound trajectories from 10 participants randomly simulated with the best-fit parameters for each model. (A) The best-fit model derived by Pinkevych et al. All participants are identical, with *k* = 1/(5.1 days). We fixed *r* = 0.4/day and fit *V*_*0*_ = 4 c/ml. (B) Allowing interperson variation in growth rate, *r*. We assumed the population distribution of *r* was log_10_-normal with log_10_-mean μ_r_ = –0.4 (10^μr^ = 0.4/day) [1,8,10–12] and fit the log_10_-standard deviation σ_r_ = 0.2 (consistent with [1,12]). We fixed *k* = 4 cells/day and fit *V*_*0*_ = 0.15 c/ml. (C) Allowing interperson variation in the activation rate, *k*. We assumed the population distribution of *k* was log_10_-normal with μ_k_ = 0.6 (10^μk^ = 4 cells/day) [1,12] and fit the log_10_-standard deviation σ_k_ = 0.55 (less than estimated in [1,12], similar to [13]). We fixed *r* = 0.4/day and fit *V*_*0*_ = 0.15 c/ml. (D) Allowing interperson variation in both activation rate and growth rate. We assumed the population distribution of *k* and *r* were log_10_-normal. Taking μ_r_ and σ_r_ to be –0.4 (10^μr^ = 0.4/day) and 0.1 and μ_k_ = 0.6 (10^μk^ = 4 cells/day), we fit σ_k_ = 0.45. We additionally fit *V*_*0*_ = 0.15 c/ml. For all simulations, the definition of viral rebound was set to 50 c/ml and the drug washout time to zero. In general, only two model parameters are identifiable from the cohort data and so the choice of which were fixed and which were fit was arbitrary. Higher *V*_*0*_ values paired with lower *r* values could fit equally well, as could either paired with higher drug washout times. Note that in simulating the Pinkevych et al. model, we allow for the possibility that multiple reactivating cells contribute to viral rebound, as otherwise the model cannot be used to describe higher activation rates.

While this model provides a good fit for rebound times (Fig 1A), it is not unique. Models allowing variation in viral dynamic parameters also fit well, and even slightly relaxing the assumption of cohort homogeneity dramatically changes the interpretation. Here, we consider three such modifications of the Pinkevych et al. model: one allowing variation in viral growth rate *r*, one allowing variation in activation rate *k*, and one allowing variation in both. Apart from this added variability, the model structure follows Pinkevych et al. exactly (see S1 Text). By demonstrating that modest levels of interperson variation alter the estimated (median) activation rate, we will conclude that rebound times alone are insufficient data for identifying the activation rate: wider interperson variation is consistent with higher activation rates, and narrower variation is consistent with lower rates. While we use an activation rate *k* = 4/day in the examples below, a range of values could be used to make this same point, and our previous work (which uses a different set of models) acknowledges uncertainty in this and related parameters [1].

First, consider variation in *r*. This rate depends on many factors, including infectivity, burst size, the density and lifespan of infectable cells, and the free virus clearance rate. These quantities can vary between individuals. Studies involving frequent viral load sampling following rebound find *r* ~ 0.4/day ± 0.2/day (sampling every 2–7 days in [8], every 2 days in [10], and every 3 days in [11,12]). Using a distribution derived from one study [12], we show that even if no other parameters vary between participants, observed rebound dynamics are consistent with frequent activation (*k* = 4/day) (Fig 1B).

The activation rate *k* can also vary between individuals. Differences in the reservoir size, the fraction containing replication-competent virus, the reactivation rate of individual cells, or the probability that each reactivated viral clone escapes stochastic extinction can produce variation in *k*. Reservoir size varies by >2 logs between infected individuals [13], and the activation state of the immune system, which influences maintenance of latency, also likely varies. Fig 1C demonstrates that this degree of variation, with frequent reactivation for the typical person (median *k* = 4/day), explains observed rebound dynamics.

Finally, if both *r* and *k* vary, there is less required variation in each (Fig 1D). Together, these three models show how variation in rebound times may be caused by many different factors and that rebound time alone is insufficient to estimate *k* when *r* or *k* vary between individuals.

Pinkevych et al. acknowledge that variation in viral dynamic parameters may exist but underestimate its importance using an underpowered statistical test for assessing the effect of this variation. One prediction of the model in which *r* varies is that rebound times are longer in participants with lower *r*. For three cohorts, the authors estimate *r* for each participant from the exponential phase of rebound. They then reason that, since the correlation between *r* and rebound time fails to reach statistical significance, variation in growth rates must be unimportant. One must be cautious, however, when employing this test using small cohorts in which statistical power for detecting a true trend may be poor. To make this point concrete, we simulated cohorts of 20 participants using the model in which *k* and *r* both vary as in Fig 1D, and we found that half of the time, no statistically significant correlation between *r* and rebound time was recovered, despite the relationship forced by the model. The true statistical power of the test is even lower: when data are censored realistically—sampling viral load only once or twice a week, estimating *r* from regression of a few points, and estimating rebound time from the first detectable viral load (cohorts 1, 2) or an interpolation (cohort 3)—then true trends are less likely to be discerned. The failure to find a trend therefore lends little confidence to the claim that variation in growth rate can be ignored in an analysis of rebound times.

Intriguingly, one of the most detailed analyses of rebound dynamics [12] does support a link between *r* and rebound time. In this study, viral loads were measured every 3 days following ART interruption and fit to a viral dynamic model using Bayesian methods. There was a significant correlation between *r* and rebound time (*R* = –0.49, *p* = 0.005). These data show another trend contradicting the assumptions of the Pinkevych et al. model. In their model, each separate treatment interruption follows the same distribution of rebound times, regardless of participants’ identity. In other words, a single participant who undergoes multiple treatment interruptions should exhibit as much variation in rebound times as a similarly sized sample of interruptions from a population of unique participants, each undergoing a single interruption. In this frequently measured cohort, however, each participant experienced three interruptions, and there were significant differences between the participants (nonparametric ANOVA, *p* = 0.02).

Pinkevych et al. present an independent analysis to support low reactivation rates that does not rely on rebound times, although it makes the same assumption of homogeneity. They use viral sequence data derived from rebounding participants in cohort 4 [9,14] to compare ratios of genetically distinguishable viral strains. They posit that if each strain behaves identically once activated, then differences in strain prevalence reflect only the different times at which each one started growing (ratio of strain 1 to strain 2 = exp(*r*(*t*_*1*_ *-t*_*2*_)). From the observed ratios, they infer the average time between activation events to be 3.6 days (assuming *r* = 0.4/day). However, an equally plausible explanation is that viral lineage frequencies vary because of fitness differences. Genetic differences between strains (on average 1.4% different [14]) may affect growth rates after activation, which can determine the observed ratios if two activate at the same time (ratio of strain 1 to strain 2 = exp((*r*_*1*_ *-r*_*2*_)t). We repeat their analysis for an alternate scenario in which strains activate simultaneously (limit of high activation rate) but grow at different rates, fitting a standard deviation of 0.09–0.19/day in growth rate (depending on the number of clones per individual (S1 Text) (Fig 2). In the context of naturally occurring HIV fitness variation, this standard deviation is not large: protease variants sampled from the same HIV-infected donor may have catalytic efficiencies varying by over 50% [15], and single drug-resistance mutations can decrease viral replication capacity by 90% [16]. Because of this variation, we do not believe that strain ratios alone can be used to estimate activation rate: as we argued previously, wider interperson variation is consistent with higher activation rates, and narrower variation is consistent with lower rates.

**Fig 2:**
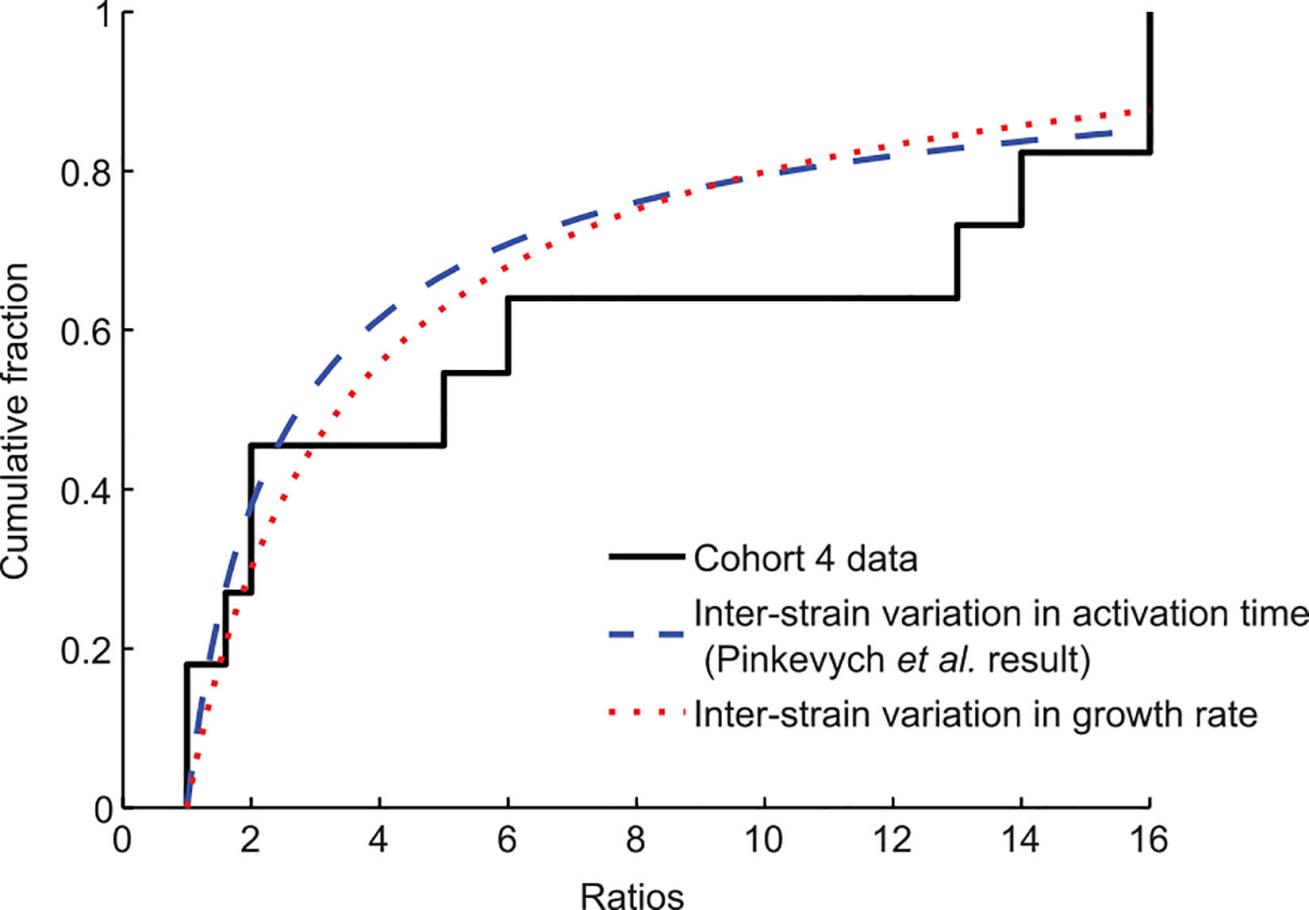
Alternatives to the Pinkevych et al. interpretation of founder virus ratios. Viral genotyping during early rebound in six participants in cohort 4 [9,14] identified multiple unique viral strains contributing to rebound and characterized their relative frequencies. Ratios were defined as the number of sequences from one strain divided by the number of sequences from the next most prevalent strain. The cumulative distribution function (CDF) for the frequency of each ratio, with all participant data combined, is shown (solid black line). Pinkevych et al. used maximum likelihood estimation to determine the activation rate *k* that best explains this distribution, assuming all strains start at the same level and grow at the same rate once reactivated. The CDF for the ratios using their estimated activation rate (*k* = 1/(3.6 days)) is shown (dashed blue line). Alternatively, we assume that strains activate at the same time (high activation rate) but that the growth rates of individual strains are normally distributed with unknown mean and variance. Using maximum likelihood estimation, we infer that an interstrain standard deviation in growth rate of 0.09/day can explain the observed clone ratios (dotted red line). This estimate increases to 0.19 under alternate assumptions about the sampling procedure (see S1 Text).

While simple models are often preferred, we believe that the foregoing discussion provides strong biological evidence against the simplifying assumption of homogeneity used in the Pinkevych et al. model. Using this model may have resulted in rate estimates considerably lower than the true values. Simplicity and realism both have merit in modeling, but the general principle behind our argument is this: even if data fits a simple model well, it is appropriate to reject conclusions that depend sensitively on the simplifying assumption if other data or biological principles contradict it.

Stepping back from the debate over modeling techniques, infrequent reactivation is at odds with aspects of clinical experience. Whether we consider the original Pinkevych et al. model, the variations presented above, or our previous model [1], if reactivation is infrequent, then time to rebound is governed by the waiting time until the first cell activation. This relationship implies that rebound time should follow the inverse of reservoir size, which seems to be contradicted by the observation that interperson variation in reservoir sizes [13,17–21] far outstrips variation in rebound times [6–11]. For the same reason, a low activation rate (less than one per viral generation) implies that any therapeutic reduction in latency is expected to prolong viral rebound, which is far more optimistic than our previous prediction that ~100-fold reduction must be achieved before substantial delays can be realized [1]. Pinkevych et al. acknowledge this point about latency reduction as well, and we hope that this discussion can spur much-needed experimental and analytical work into understanding the rate at which latently infected cells activate and fuel viral replication.

## Supporting Information

S1 Text Supplementary Methods and Results. The sources of data, model simulation, and parameter estimation are described, and results for fitting models to cohorts 1, 2, and 4 are presented.
(PDF)
