# Supplementary material for: Insufficient Evidence for Rare Activation of Latent HIV in the Absence of Reservoir-Reducing Interventions
Source: PLoS Pathog. 2016 Aug 25;12(8):e1005679. doi: 10.1371/journal.ppat.1005679 (PMC4999146; doi:10.1371/journal.ppat.1005679)
Supplement: S1 Text — The sources of data, model simulation, and parameter estimation are described, and results for fitting models to cohorts 1, 2, and 4 are presented. (PDF) [file ppat.1005679.s001.pdf]

SUPPLEMENTARY METHODS

Insufficient evidence for rare activation of latent HIV in the absence of reservoir-reducing interventions

Alison L. Hill[1, †,*], Daniel I. S. Rosenbloom[2,†], Janet D. Siliciano[3], Robert F. Siliciano[3,4]

[1] Program for Evolutionary Dynamics, Department of Mathematics, Department of Organismic and Evolutionary Biology, Harvard University, Cambridge, MA, USA
[2] Department of Biomedical Informatics, Columbia University Medical Center, New York, NY, USA
[3] Department of Medicine, Johns Hopkins University School of Medicine, Baltimore, MD, USA
[4] Howard Hughes Medical Institute, Chevy Chase, MD, USA
[†]These authors contributed equally to this work
* alhill@fas.harvard.edu

Data sources

For Cohort 3 (Figure 1), we obtained the rebound times directly from the original publication [1]. Note that these rebound times were interpolated by the original study authors by fitting an exponential curve to the first detectable viral load values. The definition of rebound was 50 copies/mL of plasma HIV RNA.

For the Cohort 4 strain ratios (Figure 2), the values for the ratios, as interpreted from the available sequence data, were not provided by Pinkevych *et al*. Therefore we used the Matlab program *grabit* to estimate data points from the figure file in their paper (Figure 5).

For the AutoVac data described in the text, we used the posterior distributions reported in Luo *et al.* [2] to estimate *k* and *r* (methods described in [3]), and we used a figure showing the rebounding dynamics for each participant to determine the time of first detectable viral load [4].

For the distribution of latent reservoir sizes discussed in the text, we used the Matlab program *grabit* to estimate data points from a figure published by Eriksson *et al.* [5].

Data for Cohort 1 and a subset of Cohort 2 patients was provided by Pinkevych *et al*. Data for Cohort 4 was obtained directly from the original publication [6].

Model simulation

We use the same model structure as in Pinkevych *et al.* The waiting time between each reactivation was an exponentially distributed random variable with mean $1/k$. Each activated strain started instantaneously at a level V_0 and then grew exponentially with rate r. When the sum of all strains reached a level V_R, rebound was declared. Note that this model has both a stochastic component (activation) and a deterministic component (growth). In the original

Pinkevych *et al.* model, only a single cell was allowed to activate and contribute to rebound. This simplification is reasonable if activation is very rare, so that by the time a second strain activates, the first strain is likely to be at a level much higher than the immediate post-activation value V_0. It is only in this limit that the proportion of number of individuals who have yet to rebound by time t is given by $S(t) = exp(-k(t-t_{delay}))$ for $t \geq t_{delay}$ and $S(t) = 1$ for $t < t_{delay}$, where t_{delay} is the combined delay for both drug washout (t_{wash}) and growth of viral load to detection level V_R ($t_{delay} = t_{wash} + \ln(V_R/V_0)/r$). However in Pinkevych *et al.*, this formula is used to infer k without putting *a priori* bounds on its size, and hence without ensuring the validity of this approximation. To avoid this problem, we directly simulated the model for all results presented, drawing exponential random variables (rate k) to determine the time between reactivation events. If multiple cells reactivated prior to viral load reaching the rebound threshold, they were all allowed to contribute to the viral population. If strain j activates after the washout period at time t_j, then it grows as $V_j(t) = V_0\, exp(r(t-t_j))$ for $t \geq t_j$. Total viral load is the sum of all V_j. There is no analytic expression for the results in this case. The “jumps” that are seen in the simulated rebound trajectories in the bottom row of Figure 1 are due to new strains activating.

Parameter estimation

In the single-activation approximation of Pinkevych *et al.*, assuming all participants have identical parameters, at most two model parameters are identifiable from cohort data. When k is small, k and t_{delay} can be uniquely identified. Of t_{wash}, V_0 and r, two must be assumed and one estimated (rebound threshold V_R is defined by experimental conditions). Using this approximation, if k is larger than about 1/day, it does not noticeably influence the time to rebound and therefore cannot be accurately estimated from cohort data.

However if multiple cells are allowed to contribute to rebound, then even when k is large enough that multiple cells reactivate each day, its value does influence the rebound time. Higher k leads to more cells activating in close succession and contributing to rebound viremia. The time of rebound will be expected to be nearly identical among all participants and only the composite parameter $t_{wash} + \ln(V_R / kV_0)/r$ can be estimated (k alone is still not identifiable).

If k or r can vary, then in the multiple-activation model, even for high k rebound time will vary between participants. Only two parameters will generally be identifiable - the composite $t_{wash} + \ln(V_R / kV_0)/r$ (which roughly determines the average rebound time) and the variance in either k or r.

Note that the lack of an analytic expression for the model when multiple activating cells contribute to rebound complicates formal identifiability analysis, and these arguments are based on approximations only.

For the results in Figure 1, we did all parameter estimation by evaluating the sum-of-squared errors for parameter values, scanning through parameters with step size of 0.05. Our goal was not to find the unique best values of parameters in these alternate scenarios, but to show that there exist values which fit the observed data as well as those suggested by Pinkevych *et al.*

We opted not to use the analytic formula for the distribution of rebound times employed by Pinkevych *et al.* for parameter inference, as this formula is specific to the limit of small k.

Since only limited parameters can be estimated from the survival curves, for the cases shown in Figure 1 we took central estimates for k and r from a previous study [2,3]. The model in that paper considered fully stochastic rebound dynamics, instead of the dual stochastic-activation-but-deterministic-growth model used by Pinkevych *et al.*, and so the comparison of model parameters is not straightforward. The previous study estimates both the total number of latent cells containing replication-competent virus that activate per day ("A"), and, the probability that any one of these cells produces a viral lineage that escapes stochastic extinction and grows large enough to contribute to rebound ("p_{Est}"). Because the Pinkevych *et al.* model only tracks activation of cells that will definitely grow, their activation rate k is equivalent to the product of A and p_{Est} from the previous study. In that study, when a latent cell reactivates the amount of virus produced (V_i) was estimated from the ratio of the viral burst size to the rate of virus clearance from plasma. This also cannot be directly compared to V_0 in the Pinkevych *et al.* model, because it is not at this low level that deterministic growth starts. Deterministic exponential growth is a valid approximation only after this initial stochastic period, when a lineage has escaped extinction. This occurs when a population has reached a size of ~1/ p_{Est} cells, and so V_0 is roughly comparable to V_i/ p_{Est} rather than V_i. The viral growth rate r and the rebound threshold V_R have the same meaning in the two models.

With these adjustments, if the Pinkevych *et al.* model is simulated allowing for multiple cells to reactivate, it will give very similar results to the former model in the limit of small reservoir reductions. For larger reservoir reductions, however, they will differ. The Pinkevych *et al.* model does not take into account the size of the reservoir and the fact that cells in it may turn over and die before reactivating. Therefore it predicts that as reservoir size is reduced – even by orders of magnitude more than the estimated number of latently infected cells – rebound will always occur, just after a longer and longer expected time. There is no option for the reservoir to be cleared by treatment or by the chance death of remaining cells, and therefore no option for patient cure – nor is this a necessary feature of the model, since its purpose is to estimate rebound times from a full-size reservoir.

Analysis of founder virus ratios

We used the same maximum likelihood framework as Pinkevych *et al.*, but instead of assuming that differences in the levels of different strains were due to different activation times, we assumed they were due to different growth rates. If two strains have growth rates r_1 and r_2, then if sampling is done t days after rebound, the expected ratio is $R=exp(\Delta r\ t)$, where $\Delta r = r_1 - r_2$. If we assume that r is normally distributed with mean μ and variance σ^2, then if only two strains are observed, r_1 and r_2, are independent and Δr is normally distributed with mean 0 and variance 2 σ^2 (the "normal difference distribution", [7]). In this study the ratios were always defined so that $\Delta r > 0$ ($R > 1$), and so $|\Delta r|$ follows the folded-normal (half-normal) distribution [8].

$$P(|\Delta r|) = \frac{1}{\sigma\sqrt{\pi}}\, e^{-\frac{\Delta r^2}{4\sigma^2}}$$

Then, using $\Delta r \equiv f(R) = ln(R)/t,$ the ratio R follows the probability density function

$$P(\mathrm{R}) = P(f(R))\frac{\partial f}{\partial R}$$
$$= \frac{1}{\sigma\sqrt{\pi}}\frac{1}{tR}e^{-\frac{\ln(\mathrm{R})^2}{4\sigma^2}}$$

and the cumulative distribution function

$$C(R) = \operatorname{erf}\left(\frac{\ln(\mathrm{R})}{2t\sigma}\right)$$

for R >1, where *erf* is the error function. If a set of N ratios R_j are measured experimentally, then the log-likelihood of the data, given the underlying distribution of growth rates (defined by σ) is $\sum_i P(R_i)$, and this function is maximized for

$$\sigma_{max} = \frac{1}{t}\sqrt{\frac{\sum_j (\ln(R_i)^2}{2N}}.$$

Because we could not find any description of the exact time after interruption for which each of these ratios were measured in any of the original papers reporting this data nor on Genbank, other than a note that it occurred "early after interruption", we assumed t=14 days (a common post-interruption sampling point), and estimated σ_{max}=0.091. If sampling was actually after only 1 week, then σ_{max}=0.182.

The above method assumes that exactly two strains are observed for each participant and used to calculate the ratios. If more strains were observed, then multiple ratios would be calculated for each individual, by ranking strains in order of their growth rates, and finding the difference between successive strains. In this case, the two growth rates being compared are no longer independent, and the expected difference between them is expected to be smaller. Because the number of strains observed differs between individuals, we cannot exactly calculate the expected distribution of R given σ. Instead, we consider two conservative scenarios that produce relatively low strain ratios and may inflate the estimated variance required to explain the data. If five strains were collected for each individual and ratios were calculated by comparing the two most frequent strains only, then using order statistics for the normal distribution [9], we numerically find that the maximum-likelihood value for σ is 0.150 (assuming sampling at 14 days). If the top two of ten strains were compared, then σ_{max} =0.186. Even in these conservative cases, the variance in r required to explain the data is in line with estimated values [1,4,10]. The cumulative distribution function curves in this case look very similar to those shown in Figure 2.

Results from other cohorts

We chose to focus on the data from Cohort 3 in the main text, because instead of reporting rebound time as the first sampling time point at which viral load was above the limit of detection (as in Cohorts 1, 2, and 4), it reported an estimate (obtained via interpolation of early viral load values) of the actual time of rebound. Although we believe a single cohort is sufficient to make

the qualitative point that the distribution in rebound times can be as easily explained by interpatient variation in parameters as by low frequency of activation of latent cells, we repeated the same analysis for the other cohorts (**Supplemental Figure 1**). The findings are very similar. Slightly more interpatient variation is required to explain rebound times in these cohorts, which is likely related to the censoring of rebound times.

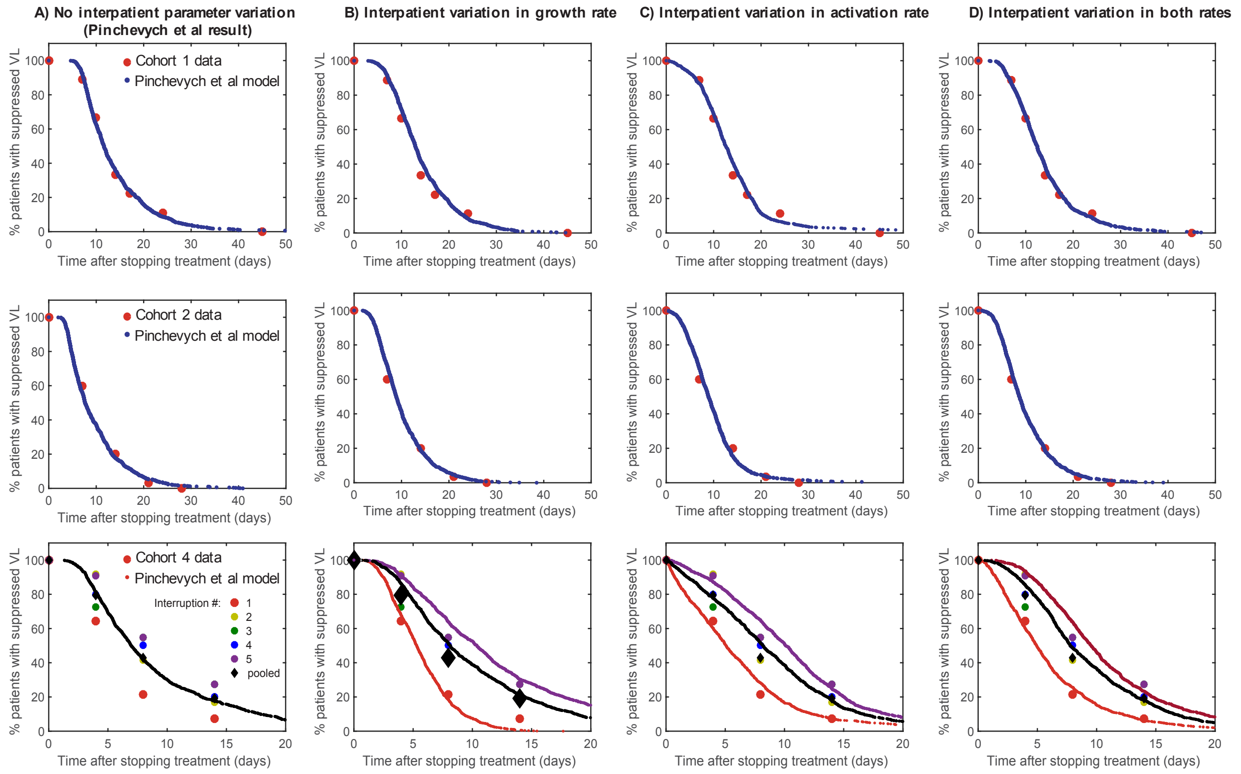

Figure S1: Alternatives to the Pinkevych *et al.* interpretation of rebound dynamics for additional cohorts. Time-to-rebound data can be explained equally well by frequent reactivation in a realistically heterogeneous cohort (Fig 1B-D) as by rare reactivation in a homogeneous population (Fig 1A). Top row: Cohort 1 [11]. Middle row: Cohort 2 [12]. Bottom row: Cohort 4 [6]. A) The best-fit models derived by Pinkevych *et al*. All participants are identical. B) Allowing inter-person variation in growth rate, *r*. We assumed the population distribution of *r* was $\log_{10}$-normal with with $\log_{10}$-mean μ_r and $\log_{10}$-standard deviation σ_r. C) Allowing inter-person variation in the activation rate, *k*. We assumed the population distribution of *k* was $\log_{10}$-normal with with $\log_{10}$-mean μ_k and $\log_{10}$-standard deviation σ_k. D) Allowing inter-person variation in both activation rate and growth rate. We assumed the population distribution of *k* and *r* were $\log_{10}$-normal. Parameters: Cohort 1 – A) k=1/(7.6 days) (fixed), r=0.4/day (fixed), V_0=3 c/ml (fit). B) μ_r=-0.4 (fixed), σ_r=0.25 (fit), k=4 cells/day (fixed), V_0=0.03 c/ml (fit). C) μ_r=-0.4 (fixed), k=4 cells/day (fixed), σ_k=1 (fit), V_0=0.03 c/ml (fit). D) μ_r=-0.4 (fixed), σ_r=0.2 (fixed), k=4 cells/day (fixed), σ_k=0.6 (fit), V_0=0.03 c/ml (fit). Cohort 2 – A) k=1/(6.3 days) (fixed), r=0.4/day (fixed), V_0=10 c/ml (fit). B) μ_r=-0.4 (fixed), σ_r=0.3 (fit), k=4 cells/day (fixed), V_0=0.15 c/ml (fit). C) μ_r=-0.4 (fixed), k=4 cells/day (fixed), σ_k=0.8 (fit), V_0=0.15 c/ml (fit). D) μ_r=-0.4 (fixed), σ_r=0.2 (fixed), k=4 cells/day (fixed), σ_k=0.6 (fit), V_0=0.15 c/ml (fit). Cohort 4 – A) k=1/(6.3 days) (fixed), r=0.4/day (fixed), V_0=15 c/ml (fit) (all interruptions pooled). B) μ_r=-0.4 (fixed), σ_r=0.4 (fit), k=4 cells/day (fixed), V_0=0.1-0.7 c/ml (fit) (varies by interruption number). C) μ_r=-0.4 (fixed), k=4 cells/day (fixed), σ_k=1 (fit), V_0=0.1-0.7 c/ml (fit) (varies by interruption number). D) μ_r=-

0.4 (fixed), σ_r=0.2 (fixed), k=4 cells/day (fixed), σ_k=0.7 (fit), V_0=0.1-0.7 c/ml (fit) (varies by interruption number).

References

1. Davey, Jr. RT, Bhat N, Yoder C, Chun T-W, Metcalf JA, Dewar R, et al. HIV-1 and T cell dynamics after interruption of highly active antiretroviral therapy (HAART) in patients with a history of sustained viral suppression. Proc Natl Acad Sci USA. 1999;96: 15109–15114.

2. Luo R, Piovoso MJ, Martinez-Picado J, Zurakowski R. HIV Model Parameter Estimates from Interruption Trial Data including Drug Efficacy and Reservoir Dynamics. PLoS ONE. 2012;7: e40198. doi:10.1371/journal.pone.0040198

3. Hill AL, Rosenbloom DIS, Fu F, Nowak MA, Siliciano RF. Predicting the outcomes of treatment to eradicate the latent reservoir for HIV-1. Proc Natl Acad Sci. 2014;111: 13475–13480. doi:10.1073/pnas.1406663111

4. Ruiz L, Carcelain G, Martínez-Picado J, Frost S, Marfil S, Paredes R, et al. HIV dynamics and T-cell immunity after three structured treatment interruptions in chronic HIV-1 infection. Aids. 2001;15: F19–F27.

5. Eriksson S, Graf EH, Dahl V, Strain MC, Yukl SA, Lysenko ES, et al. Comparative Analysis of Measures of Viral Reservoirs in HIV-1 Eradication Studies. PLoS Pathog. 2013;9: e1003174. doi:10.1371/journal.ppat.1003174

6. Fischer M, Hafner R, Schneider C, Trkola A, Joos B, Joller H, et al. HIV RNA in plasma rebounds within days during structured treatment interruptions. AIDS Lond Engl. 2003;17: 195–199. doi:10.1097/01.aids.0000042945.95433.4b

7. Weisstein EW. Normal Difference Distribution [Internet]. [cited 4 May 2016]. Available: http://mathworld.wolfram.com/NormalDifferenceDistribution.html

8. Leone FC, Nelson LS, Nottingham RB. The Folded Normal Distribution. Technometrics. 1961;3: 543–550. doi:10.2307/1266560

9. Gupta SS. Percentage Points and Modes of Order Statistics from the Normal Distribution. Ann Math Stat. 1961;32: 888–893. doi:10.1214/aoms/1177704982

10. Ruiz L, Martinez-Picado J, Romeu J, Paredes R, Zayat MK, Marfil S, et al. Structured treatment interruption in chronically HIV-1 infected patients after long-term viral suppression. AIDS. 2000;14: 397.

11. Rasmussen TA, Tolstrup M, Brinkmann CR, Olesen R, Erikstrup C, Solomon A, et al. Panobinostat, a histone deacetylase inhibitor, for latent-virus reactivation in HIV-infected patients on suppressive antiretroviral therapy: a phase 1/2, single group, clinical trial. Lancet HIV. 2014;1: e13–e21.

12. Bloch MT, Smith DE, Quan D, Kaldor JM, Zaunders JJ, Petoumenos K, et al. The role of hydroxyurea in enhancing the virologic control achieved through structured treatment

interruption in primary HIV infection: final results from a randomized clinical trial (Pulse). J Acquir Immune Defic Syndr. 2006;42: 192–202. doi:10.1097/01.qai.0000219779.50668.e6
